# Diretriz Brasileira de Reabilitação Cardiovascular: Valores e Limitações

**DOI:** 10.36660/abc.20200995

**Published:** 2020-12-14

**Authors:** Marlus Karsten, Ariany Marques Vieira, Gabriela Lima de Melo Ghisi

**Affiliations:** 1 Universidade do Estado de Santa Catarina Programa de Pós-graduação em Fisioterapia FlorianópolisSC Brasil Universidade do Estado de Santa Catarina - Programa de Pós-graduação em Fisioterapia, Florianópolis, SC - Brasil; 2 Universidade Federal de Ciências da Saúde de Porto Alegre Programa de Pós-graduação em Ciências da Reabilitação Porto AlegreRS Brasil Universidade Federal de Ciências da Saúde de Porto Alegre - Programa de Pós-graduação em Ciências da Reabilitação,Porto Alegre, RS - Brasil; 3 Concordia University Department of Health, Kinesiology, and Applied Physiology MontrealQuebec Canadá Concordia University - Department of Health, Kinesiology, and Applied Physiology, Montreal, Quebec - Canadá; 4 Montreal Behavioural Medicine Centre MontrealQuebec Canadá Montreal Behavioural Medicine Centre - CIUSSS du Nord-de-l’Île-de-Montréal, Montreal, Quebec - Canadá; 5 University Health Network Cardiovascular Prevention and Rehabilitation Program TorontoOntario Canadá University Health Network - Cardiovascular Prevention and Rehabilitation Program, Toronto, Ontario – Canadá

**Keywords:** Doenças cardiovasculares, Prevenção secundária, Reabilitação, Exercício, Educação em saúde, Profissionais de saúde

Prezado editor,

A Diretriz Brasileira de Reabilitação Cardiovascular – 2020^1^é de grande interesse e relevância aos profissionais envolvidos na assistência a pacientes com doença cardiovascular (DCV). Com componentes principais internacionalmente acordados,^2^a reabilitação cardiovascular (RCV) é um modelo bem estabelecido de prevenção secundária que atenua a carga das DCVs. Apesar de seus benefícios, a RCV está disponível em apenas 40% dos países de baixa renda, com capacidade insuficiente até nos lugares onde existe^3^. Dessa forma, diretrizes para profissionais de saúde em países onde a RCV não está amplamente disponível são extremamente necessárias.

Embora a diretriz apresente melhorias conceituais em relação às versões anteriores, alguns pontos merecem maior atenção. Primeiro, o título se refere à RCV no geral e, uma vez que o documento foca em treinamento de exercícios, ele não aborda, de forma detalhada, outros componentes essenciais da RCV, nem recursos relevantes de avaliação e prescrição de exercícios.^4^ Embora seja amplamente conhecido que o exercício é a base da RCV, o manejo de pacientes com DCV é multifatorial, incluindo não apenas exercícios, mas também processos de educação do paciente, promoção de mudança comportamental, apoio psicossocial, aconselhamento nutricional, otimização do tratamento farmacológico, estratégias de cessação do tabagismo etc. Programas abrangentes de RCV (ou seja, que incluem exercícios combinados com todos os componentes mencionados acima) fornecem benefícios adicionais aos pacientes, incluindo redução nas taxas de mortalidade por todas as causas. No Brasil, o primeiro ensaio clínico randomizado em um país de baixa e média renda concluiu que a RCV abrangente pode melhorar os resultados clínicos, os comportamentos relacionados à saúde cardiovascular e o conhecimento sobre a doença, bem como diminuir a morbidade, com manutenção dos ganhos por 1 ano^5^.

Uma abordagem multifatorial na assistência a pacientes com DCV é alcançada com uma equipe multiprofissional (médicos, fisioterapeutas, enfermeiras, nutricionistas, profissionais de educação física, psicólogos etc.). É necessário levar em consideração e valorizar todos os profissionais de saúde envolvidos na assistência a pacientes em RCV, ou seja, os profissionais que possibilitam a execução efetiva desses programas. A complexidade dos problemas de pacientes com DCV é um bom exemplo da necessidade real do uso de uma abordagem de equipe que envolva diferentes disciplinas, especialidades e habilidades. Além disso, é importante considerar a estrutura histórica da RCV no Brasil, em que as equipes multidisciplinares e o papel autônomo que cada profissional pode exercer são desvalorizados. A centralização do processo de RCV pode acabar sendo mais uma barreira, junto a tantas outras existentes no nosso país, como a falta de financiamento.

A estruturação de programas de RCV de acordo com a estratificação de risco e a certificação profissional podem representar uma nova e promissora etapa que acelera uma transição metodológica^6^. Instituições brasileiras e internacionais estão em constante desenvolvimento no que diz respeito à RCV multidisciplinar e podem compartilhar esforços para melhorar a disponibilidade e eficácia dos programas, reduzindo a possível sobrecarga para os cardiologistas, que geralmente estão envolvidos em outras atribuições profissionais.

É importante ressaltar que as diretrizes visam influenciar os profissionais de saúde, fornecendo suporte baseado em evidências para que os tomadores de decisão possam melhorar a qualidade da assistência. De acordo com o *AGREE Consortium* ^7^, os benefícios das diretrizes estão relacionados à qualidade dos próprios documentos, como seu escopo e propósito, o envolvimento das partes interessadas e o rigor de desenvolvimento, que estão principalmente relacionados a uma abordagem sistemática, bem como à sua clareza de apresentação. Alguns desses domínios não estão claros na Diretriz Brasileira de Reabilitação Cardiovascular – 2020. O envolvimento das partes interessadas e a abordagem sistemática em relação às evidências científicas, por exemplo, devem ser cuidadosamente reconsiderados na próxima versão.

## References

[1] . Carvalho T, Milani M, Ferraz AS, Silveira AS, Herdy AH, Horssi CAC, et al. Diretriz Brasileira de Reabilitação Cardiovascular-2020. Arq Bras Cardiol. 2020; 114(5):943-87. 10.36660/abc.20200407 PMC8387006 32491079

[2] . Grace S, Turk-Adawi KI, Contractor A, Atrey A, Campbell NRC, Derman W, et al. Cardiac Rehabilitation Delivery Model for Low-Resource Settings: An International Council of Cardiovascular Prevention and Rehabilitation Consensus Statement. Prog Cardiovasc Dis. 2016; 59(3):303-22. 10.1016/j.pcad.2016.08.004 27542575

[3] . Pesah E, Turk-Adawi K, Supervia M, Lopez-Jimenez F, Britto R, Ding RA, et al. Cardiac rehabilitation delivery in low/middle-income countries, Heart. 2019; 105:1806–12. 10.1136/heartjnl-2018-314486 31253695

[4] . van Halewijn G, Deckers J, Tay HY, van Domburg R, Kotseva K, Wood D. Lessons from contemporary trials of cardiovascular prevention and rehabilitation: A systematic review and meta-analysis. Int J Cardiol. 2017; 232:294-303. 10.1016/j.ijcard.2016.12.125 28094128

[5] . Chaves GSS, Ghisi GLM, Grace SL, Oh P, Ribeiro AL, Britto RR. Effects of comprehensive cardiac rehabilitation on functional capacity in a middle-income country: a randomized trial. Heart. 2019; 105:406–13. 10.1136/heartjnl-2018-313632 30282639

[6] . Scherrenberg M, Wilhelm M, Hansen D, Völler H, Cornelissen V, Frederix I, et al. The future is now: a call for action for cardiac telerehabilitation in the COVID-19 pandemic from the secondary prevention and rehabilitation section of the European Association of Preventive Cardiology. Eur J Prev Cardiol. 2020 Jul 02;2047487320939671 ahead of print 10.1177/2047487320939671 PMC7928994 32615796

[7] . AGREE Next Steps Consortium (2009). The AGREE II Instrument [Electronic version]. Retrieved July, 07, 2020 Available from: http://www.agreetrust.org.

