# Supplementary material for: Diretriz Brasileira de Reabilitação Cardiovascular: Valores e Limitações
Source: Arq Bras Cardiol. 2020 Dec 14;115(6):1208–9. [Article in Portuguese] doi: 10.36660/abc.20200995 (PMC8133740; doi:10.36660/abc.20200995)
Supplement: Translation

# Translation: Brazilian Cardiovascular Rehabilitation Guideline: Values and Limitations

**Authors:** Marlus Karsten, Ariany Marques Vieira, Gabriela Lima de Melo Ghisi

**Affiliations:** 1 Brazil Universidade do Estado de Santa Catarina - Programa de Pós-graduação em Fisioterapia, Florianópolis, SC - Brazil; 2 Brazil Universidade Federal de Ciências da Saúde de Porto Alegre - Programa de Pós-graduação em Ciências da Reabilitação,Porto Alegre, RS - Brazil; 3 Canada Concordia University - Department of Health, Kinesiology, and Applied Physiology, Montreal, Quebec - Canada; 4 Canada Montreal Behavioural Medicine Centre - CIUSSS du Nord-de-l’Île-de-Montréal, Montreal, Quebec - Canada; 5 Canada University Health Network - Cardiovascular Prevention and Rehabilitation Program, Toronto, Ontario – Canada

**Keywords:** Cardiovascular Diseases, Secondary prevention, Reabilitation, Exercise, Health Education, Health Care Providers

Dear Editor,

The Brazilian Cardiovascular Rehabilitation Guideline – 2020^1^is of great interest and relevance to the professionals involved in the care of cardiovascular disease (CVD) patients. With internationally agreed core components,^2^cardiovascular rehabilitation (CR) is a well-established model of secondary prevention that mitigates the CVD burden. Despite its benefits, the CR is available in only 40% of low-middle income countries, with insufficient capacity even where it does exist.^3^Therefore, guidelines for healthcare providers in countries where CR is not widely available are sorely needed.

Although these guidelines show conceptual improvements over previous versions, some points deserve greater attention. First, the title refers to CR in general and, since the document focuses on exercise training it fails to address other important CR core components in detail, as well as relevant features of exercise assessment and prescription. Although it is widely known that the cornerstone of CR is exercise, the management of CVD patients is multifactorial, including not only exercise but patient education, promoting behavior change, psychosocial support, nutrition counseling, optimizing medication, smoking cessation strategies, etc. Comprehensive CR programs (i.e., exercise combined with all of the aforementioned components) have provided additional benefits to patients, including reduction in all-cause mortality rates.^4^In Brazil, the first-ever randomized controlled trial in a low-and-middle income country confirmed that comprehensive CR can improve clinical outcomes, heart health behaviors, and disease-related knowledge, as well as decrease morbidity, with 1-year maintenance of gains.^5^

A multifactorial approach for the care of CVD patients is achieved with a multi-professional team (doctors, physiotherapists, nurses, dietitians, physical education professionals, psychologists, etc.). It is necessary to consider and value all of the healthcare providers involved in the care of CR patients, i.e., those who enable these programs to run and be effective. The complexity of CVD patients’ problems is a good example of the real need to use a team approach that involves different disciplines, expertise, and skills. It is also important to consider the historical structure of CR in Brazil, where multidisciplinary teams and the autonomous role that each professional can play are undervalued. Centralization of the CR process may turn out to be one more barrier, along with the many others in our country, such as lack of funding.

Structuring CR programs according to risk stratification and professional certification can represent a new and promising stage that hastens a methodological transition.^6^Brazilian and international institutions are in constant development regarding multidisciplinary CR and may share efforts to improve the availability and effectiveness of programs, reducing the possible burden on cardiologists, who are usually engaged in other professional assignments.

It is worth noting that guidelines aim to influence healthcare professionals, providing evidence-based support so that decision makers can improve the quality of care. According to the AGREE Consortium,^7^the benefits of guidelines are related to the quality of the documents themselves, such as their scope and purpose, stakeholder involvement, and rigor of development, which is mainly related to a systematic approach, as well as their clarity of presentation. Some of these domains are not clear in the Brazilian Cardiovascular Rehabilitation Guideline – 2020. Stakeholder involvement and a systematic approach toward scientific evidence, for example, must be carefully reconsidered in the next version.

# Translation: Reply

First of all, we would like to thank the authors of the letter “Brazilian Cardiovascular Rehabilitation Guidelines: values and limitations” for their interest in the Brazilian Cardiovascular Rehabilitation Guideline – 2020,^1^for recognizing that there has been a conceptual evolution in relation to the previous versions of our guidelines and positions and, particularly, for their criticisms, which provided us with a unique opportunity to resolve questions and address topics of great relevance, many of which were not included in the document due to authors’ decisions, as will be justified below.

We agree “ *ipsis literis* ” with the statement that one of the biggest problems with cardiovascular rehabilitation (CR) worldwide is the scarcity of structured programs,^2^including in Brazil,^3^where, as we emphasize in our document, the chronic situation calls for urgent public health strategies to make CR viable in both public (SUS) and private (ANS) health systems, since it is clearly an important health policy issue that must be resolved.^1 , 3 , 4^

We also agree that guidelines are needed to influence health professionals. In this context, we should point out that the Brazilian Society of Cardiology (SBC) has been doing so for more than two decades,^4^i.e. the current guideline is the sixth such document to specifically address CR.^1 , 4 - 8^We should also add that the two SBC prevention guidelines,^9 , 10^in addition to physical exercise, addressed other topics such as global prevention strategies, which obviously must be considered in the context of CR.

Other health professionals have even participated in some of these SBC documents,^4 , 5 , 9 , 10^which shows our recognition of and strong commitment to an interdisciplinary, multi-professional approach to make CR more effective.^11^We are unaware of other guidelines or positions on CR in Brazil by other societies or associations of health professionals. Therefore, this is an important gap to be filled, including discussion about the performance of each professional in the interdisciplinary approach.

Although the title of the current guideline refers to CR in general, the introduction clearly states that “as in previous documents the Brazilian Society of Cardiology […] has published on the subject,^4 - 8^the guideline exclusively addresses interventions based on physical exercise aimed at treating patients with cardiovascular diseases, with the class (or degree) of recommendation always based on the highest available level of scientific evidence”.^1^

Thus, the main objective of the current guideline was clearly defined, although this obviously does not mean disregarding a broader approach to a structured change of behavior. Promoting a healthy lifestyle includes patient education processes, psychosocial support, nutritional counseling, optimization of and adherence to pharmacological treatment, smoking cessation strategies, strategies for modulating stress, etc. This requires multiprofessional participation and an interdisciplinary approach, which, as explained above, has been duly considered in other SBC guidelines and positions on prevention, the latest update being 2019 document.^10^

In addition to the reduced availability, the inclusion and treatment adherence of those eligible for CR services are low.^11^We consider that, particularly in Brazil, one of the main barriers to CR is the lack referral by attending physicians,^12^including the “fear” of exercise for more severe patients. This denotes ignorance about CR, despite consistent and well-documented publications on its safety and efficacy, specifically its reduction of morbidity, mortality and hospitalization, combined with increased quality of life.^13 , 14^

Therefore, the main goal of the Brazilian Cardiovascular Rehabilitation Guideline – 2020^1^was to update information about the method, emphasizing exercise programs and the importance of more active participation by attending physicians and members of CR programs. Thus, we focused the search for scientific evidence on the indications and benefits of CR, especially regarding exercise as a treatment for cardiovascular diseases.

The current Guideline cites 382 references, practically half of which were published in the last five years, which shows our concern with using current scientific evidence to assess the impact of physical activity on numerous diseases and clinical situations, not merely coronary disease and heart failure. The effects of exercise were described in detail, enabling safe and effective prescription in individuals with hypertrophic cardiomyopathy, arrhythmogenic right ventricular cardiomyopathy, myocarditis, valvulopathy, heart transplantation, implantable cardiac devices and peripheral obstructive arterial disease, for example.^1^

In view of the above, we would like to emphasize that:

1. The Brazilian Cardiovascular Rehabilitation Guideline – 2020 was developed by members of the SBC, i.e. a medical society. Twenty-two experienced authors from different public and private services in various regions of Brazil worked on the CR and, as previously mentioned, its main goal was to update and expand information about the impact of exercise on patients with cardiovascular disease, aiming to value physician intervention and increase patient referrals to formal CR services.
2. Although our focus on multiprofessional performance was purposely limited, it was not treated as irrelevant. We stated that “the multiprofessional team usually consists of doctors, physical educators, physical therapists and nursing professionals. Other professionals can also be included in the team, such as nutritionists, psychologists and social workers”. We also explained that “like physicians, when the other members of the team perform their respective functions, they must follow the norms and rules that guide the program, respecting the recommendations of their respective professional councils”.
3. In Brazil, we believe that more direct participation by medical professionals is still necessary, unlike in other countries, such as Canada, since the decision to include CR in the context of full clinical treatment is initially up to the attending physician.
4. The SBC guidelines have always considered stakeholders, historically enabling more effective and competent performance for the benefit of patients, as well as providing effective measures for the competent bodies (ANS and SUS) to define therapeutic strategies. We believe that the latter should be one consequences of this document.

**Tales de Carvalho**

**Mauricio Milani**

**Coordinators of the Brazilian Cardiovascular Rehabilitation Guideline – 2020**
