# Supplementary material for: Diretriz Brasileira de Reabilitação Cardiovascular: Valores e Limitações
Source: Arq Bras Cardiol. 2020 Dec 14;115(6):1208–9. [Article in Portuguese] doi: 10.36660/abc.20200995 (PMC8133740; doi:10.36660/abc.20200995)
Supplement: Related texts

# Reply: Carta-resposta

Primeiramente, agradecemos aos autores da carta ao editor, intitulada *“Brazilian Cardiovascular Rehabilitation Guidelines: values and limitations”* , pelo interesse na Diretriz Brasileira de Reabilitação Cardiovascular 2020,^1^pelo reconhecimento de que houve uma evolução conceitual em relação às versões anteriores de nossas diretrizes e posicionamentos e, particularmente, pelas críticas que nos proporcionaram a oportunidade única de dirimir dúvidas e abordar alguns temas de grande relevância, muitos dos quais não foram contemplados no documento por decisão dos próprios autores, conforme será justificado adiante.

Concordamos *ipsis litteris* com a afirmação de que um dos maiores problemas da reabilitação cardiovascular (RCV) é a escassez de programas estruturados globalmente,^2^inclusive no Brasil,^3^onde, conforme enfatizamos no nosso documento, existe uma situação crônica que clama por uma adoção urgente de estratégias de saúde pública que viabilizem a RCV nos sistemas público [via Sistema Único de Saúde (SUS)] e privado de saúde [via Agência Nacional de Saúde Suplementar (ANS)], por se tratar claramente de uma importante questão de política de saúde a ser resolvida.^1 , 3 , 4^

Também concordamos com a afirmação de que são necessárias diretrizes para orientar os profissionais de saúde. Nesse contexto, devemos ressaltar que, no Brasil, a Sociedade Brasileira de Cardiologia (SBC) vem se posicionando há mais de duas décadas,^4^sendo a atual diretriz o sexto documento que abordou especificamente a RCV.^1 , 4 - 8^Devemos acrescentar à essa relação as duas diretrizes de prevenção da SBC,^9 , 10^nas quais, além do exercício físico, são abordados com destaque os demais temas propostos, como estratégias globais de prevenção, que obviamente devem ser considerados no contexto da RCV.

Alguns desses documentos da SBC têm, inclusive, contado com a relevante participação dos demais profissionais de saúde,^4 , 5 , 9 , 10^comprovando o reconhecimento e o forte comprometimento com a necessária abordagem interdisciplinar, que exige participação multiprofissional, com o intuito de que a RCV se torne mais efetiva.^11^Infelizmente, desconhecemos a publicação de diretrizes e posicionamentos sobre a RCV no Brasil elaboradas por outras sociedades ou associações de profissionais de saúde, o que consideramos ser uma importante lacuna a ser preenchida, inclusive para gerar uma consequente discussão sobre a atuação de cada profissional na abordagem interdisciplinar que se faz necessária.

Embora o título da atual diretriz se refira à RCV em geral, já na introdução do documento consta que “a exemplo do que ocorreu nos documentos anteriormente publicados pela Sociedade Brasileira de Cardiologia (SBC) sobre o tema,^4 - 8^a diretriz aborda exclusivamente a intervenção com base na prática de exercícios físicos direcionados ao tratamento de pacientes com doenças cardiovasculares, sendo a classe (ou grau) de recomendação sempre fundamentada no mais elevado nível de evidência científica encontrado”.^1^

Desse modo, o principal objetivo da atual diretriz foi definido de forma clara, mas isso não significa que foi desconsiderada a relevância de uma abordagem ampla, em prol de uma mudança estruturada de comportamento, com adoção de um estilo de vida saudável, incentivado por meio de processos de educação do paciente, suporte psicossocial, aconselhamento nutricional, otimização e adesão ao tratamento farmacológico, estratégias de cessação do tabagismo, estratégias para modulação do estresse etc. Para isso, é necessária participação multiprofissional em abordagem interdisciplinar, que, conforme já explicitado, tem sido devidamente considerada em outras diretrizes e posicionamentos sobre prevenção da própria SBC, sendo a última atualização o documento publicado em 2019.^10^

Além da reduzida disponibilidade de serviços de RCV já citada, há também uma baixa inclusão e adesão dos pacientes elegíveis aos serviços disponíveis.^11^Consideramos que, particularmente no Brasil, uma das principais barreiras seja a falta de orientação e encaminhamento pelo médico assistente,^12^inclusive pelo “temor” ao exercício direcionado a pacientes mais graves. Isso denota desconhecimento sobre a RCV, apesar das consistentes e bem documentadas publicações a respeito de sua segurança e eficácia, que refletem em reduções da morbimortalidade e da taxa de hospitalização, aliadas ao expressivo ganho de qualidade de vida.^13 , 14^

Portanto, a Diretriz Brasileira de Recuperação Cardiovascular 2020^1^estabeleceu como principal meta atualizar as informações sobre o método, com ênfase nos programas de exercícios físicos e na importância de uma participação mais ativa dos médicos assistentes e integrantes dos programas de RCV. Sendo assim, a busca por evidências científicas se concentrou nas indicações e nos benefícios da RCV com ênfase no exercício físico como tratamento das doenças cardiovasculares.

A atual Diretriz possui 382 referências citadas, sendo praticamente metade composta por artigos publicados nos últimos 5 anos. Isso evidencia a preocupação com uma abordagem embasada em evidência científica atualizada, tendo como objetivo abordar em detalhe o impacto da atividade física em um grande número de doenças e situações clínicas, não se restringindo apenas à doença coronária e à insuficiência cardíaca de um modo geral. Nesse contexto, foram descritos detalhadamente os efeitos dos exercícios físicos, possibilitando a sua segura e efetiva prescrição, em indivíduos com miocardiopatia hipertrófica, cardiopatia arritmogênica do ventrículo direito, miocardite, valvulopatias, transplante cardíaco, dispositivos cardíacos implantáveis e doenças arteriais obstrutivas periféricas, por exemplo.^1^

Tendo em vista o exposto, gostaríamos de ressaltar que:

1. A Diretriz Brasileira de Recuperação Cardiovascular 2020 foi desenvolvida por membros da SBC, com 22 autores experientes e atuantes na RCV em diferentes serviços públicos e privados de várias regiões do Brasil e, conforme já mencionado, teve como principal meta atualizar e aprofundar as informações sobre o impacto do exercício físico em pacientes com doença cardiovascular, visando à valorização da intervenção pelos médicos, com incremento de encaminhamento dos pacientes aos serviços formais de RCV.
2. A abordagem da atuação multiprofissional foi propositadamente limitada, mas não considerada irrelevante, constando no texto do documento que “a equipe multiprofissional habitualmente é composta por médicos, educadores físicos, fisioterapeutas e profissionais de enfermagem. Outros profissionais também podem compor a equipe, como nutricionistas, psicólogos e assistentes sociais”, estando também explicitado que, “de modo semelhante aos médicos, os demais membros da equipe, ao executarem suas respectivas funções, devem seguir as normas e regras que norteiam as atividades do programa, respeitando as recomendações de seus respectivos conselhos profissionais”.
3. No Brasil, ainda julgamos necessária uma participação mais direta dos profissionais médicos, ao contrário do que ocorre em outros países, como o Canadá, pois a decisão de incluir a RCV no contexto do tratamento clínico pleno cabe inicialmente ao médico assistente.
4. As diretrizes da SBC têm sempre levado em consideração as partes interessadas ( *stakeholders* ), historicamente possibilitando atuação mais efetiva e competente de profissionais em benefício dos pacientes e auxiliando na obtenção de medidas efetivas dos órgãos competentes (ANS e SUS) para a definição de estratégias terapêuticas. Acreditamos que essa última situação deva ser uma das consequências deste documento.

**Tales de Carvalho**

**Mauricio Milani**

**Coordenadores da Diretriz Brasileira de Reabilitação Cardiovascular 2020**

## References

[1] . Carvalho T, Milani M, Ferraz AS, Silveira ADD, Herdy AH, Hossri CAC, et al. Brazilian Cardiovascular Rehabilitation Guideline - 2020. Arq Bras Cardiol. 2020;114(5):943-87.10.36660/abc.20200407PMC838700632491079

[2] . Turk-Adawi K, Supervia M, Lopez-Jimenez F, Pesah E, Ding R, Britto RR, et al. Cardiac Rehabilitation Availability and Density around the Globe. EClinicalMedicine. 2019;13:31-45.10.1016/j.eclinm.2019.06.007PMC673720931517261

[3] . Britto RR, Supervia M, Turk-Adawi K, Chaves G, Pesah E, Lopez-Jimenez F, et al. Cardiac rehabilitation availability and delivery in Brazil: a comparison to other upper middle-income countries. Braz J Phys Ther. 2020;24(2):167-76.10.1016/j.bjpt.2019.02.011PMC708267530862431

[4] . Godoy M. I Consenso Nacional de Reabilitação Cardiovascular. Arq Bras Cardiol. 1997;69(4):267-91.9595722

[5] . Araujo CG, Carvalho T, Castro CL, Costa RV, Moraes RS, Oliveira Filho JA, et al. Normatização dos Equipamentos e Técnicas da Reabilitação Cardiovascular Supervisionada. Arq Bras Cardiol. 2004;83(5):448-52.10.1590/s0066-782x200400170001215543365

[6] . Moraes RS, Nobrega AC, Castro RRT, Negrão CE, Stein R, Serra SM, et al. Diretriz de Reabilitação Cardíaca. Arq Bras Cardiol. 2005;84(5):431-40.10.1590/S0066-782X200500050001515917979

[7] . Carvalho T, Cortez AA, Ferraz A, Nóbrega ACL, Brunetto AF, Herdy AH, et al. Diretriz de Reabilitação cardiopulmonar e meatabólica: aspectos práticos e responsabilidades. Arq Bras Cardiol. 2006;86(1):74-82.10.1590/s0066-782x200600010001116491212

[8] . Herdy AH, Lopez-Jimenez F, Terzic CP, Milani M, Stein R, Carvalho T, et al. Consenso Sul-Americano de Prevenção e Reabilitação Cardiovascular. Arq Bras Cardiol. 2014;103(2 Suppl 1):1-31.10.5935/abc.2014s00325387466

[9] . Simao AF, Precoma DB, Andrade JP, Correa FH, Saraiva JF, Oliveira GM, et al. [I Brazilian Guidelines for cardiovascular prevention]. Arq Bras Cardiol. 2013;101(6 Suppl 2):1-63.10.5935/abc.2013S01224554026

[10] . Precoma DB, Oliveira GMM, Simao AF, Dutra OP, Coelho OR, Izar MCO, et al. Updated Cardiovascular Prevention Guideline of the Brazilian Society of Cardiology - 2019. Arq Bras Cardiol. 2019;113(4):787-891.10.5935/abc.20190204PMC702087031691761

[11] . Ruano-Ravina A, Pena-Gil C, Abu-Assi E, Raposeiras S, van ‘t Hof A, Meindersma E, et al. Participation and adherence to cardiac rehabilitation programs. A systematic review. Int J Cardiol. 2016;223:436-43.10.1016/j.ijcard.2016.08.12027557484

[12] . Daly J, Sindone AP, Thompson DR, Hancock K, Chang E, Davidson P. Barriers to participation in and adherence to cardiac rehabilitation programs: a critical literature review. Prog Cardiovasc Nurs. 2002;17(1):8-17.10.1111/j.0889-7204.2002.00614.x11872976

[13] . Anderson L, Oldridge N, Thompson DR, Zwisler AD, Rees K, Martin N, et al. Exercise-Based Cardiac Rehabilitation for Coronary Heart Disease: Cochrane Systematic Review and Meta-Analysis. J Am Coll Cardiol. 2016;67(1):1-12.10.1016/j.jacc.2015.10.04426764059

[14] . Taylor RS, Sagar VA, Davies EJ, Briscoe S, Coats AJ, Dalal H, et al. Exercise-based rehabilitation for heart failure. Cochrane Database Syst Rev. 2014(4):CD003331.10.1002/14651858.CD003331.pub4PMC648590924771460
